# The scent of senescence: Age-dependent changes in the composition of the cephalic gland secretion of the male European beewolf, Philanthus triangulum

**DOI:** 10.1673/2006_06_20.1

**Published:** 2006-09-22

**Authors:** Martin Kaltenpoth, Erhard Strohm

**Affiliations:** 1 University of Würzburg, Department for Animal Ecology and Tropical Biology, Am Hubland, 97074 Würzburg; 2 University of Regensburg, Department of Zoology, 93040 Regensburg

**Keywords:** Communication, aging, mate choice, sexual selection, Hymenoptera, Crabronidae

## Abstract

The process of aging inevitably leads to changes in physiology, performance and fertility of eukaryotic organisms and results in trade-offs in the resource allocation between current and future reproduction and longevity. Such constraints may also affect the production of complex and costly signals used for mate attraction and might therefore be important in the context of mate choice. We investigated age-related changes in the amount and composition of the cephalic gland secretion that male European beewolves, Philanthus triangulum (Hymenoptera, Crabronidae) use to mark their territories. The secretion mainly consists of eleven long-chain compounds with large proportions of a carbon acid, a ketone and two alcohols, and small proportions of several alkanes and alkenes. Both the total amount and the composition of the gland content varied with age. The four compounds with functional groups were present in much lower proportions in very young and very old males compared to middle-aged males, suggesting that these components may be more costly than the alkanes and alkenes. Thus, physiological constraints may cause the delayed onset and early decline of these substances in the cephalic gland. There were also minor but significant changes in four components among the middle-aged males. These age-related changes in the amount and composition of the male marking secretion might provide reliable indicators for female choice.

## Introduction

Senescence is a process that inevitably affects all higher organisms. The continuous accumulation of deleterious mutations, changes in protein synthesis, oxidative damage in mitochondria, and the accumulation of harmful metabolic end-products all contribute to the aging process and result in a finite life span (Collatz et al. 1986). The restricted life time selects for an optimal allocation of limited resources to reproduction and survival in order to maximize fitness (Roff 1992; Stearns 1992). Several studies have demonstrated the resulting trade-offs between current and future reproduction and longevity (Partridge and Farquhar 1981; Prowse and Partridge 1997).

Considering the trade-offs in resource allocation between reproduction and longevity, age is often regarded as an important factor for mate choice. Decreasing viability and fertility (quality and/or amount of sperm) and the accumulation of deleterious germ-line mutations throughout the lifetime might contribute to a negative relationship between age and mate quality (Hansen and Price 1995; Brooks and Kemp 2001). As a consequence, females are often expected to prefer younger males (Hansen and Price 1995;Brooks and Kemp 2001). By contrast, many authors argue that females should prefer older mates because they have already demonstrated their viability and might therefore provide good genes for the offspring (Trivers 1972; Manning 1985). Thus, although it is generally accepted that age may be an important factor influencing mate choice, the direction of the preference is still a matter of debate and is likely to vary among organisms.

In animals where age plays a role for mate choice, indicator mechanisms must be available that allow the assessment of a potential mate’s age. Many insects heavily rely on pheromones for inter- and intraspecific communication. Although in most insect taxa, sex pheromones are produced by females, male sex pheromones occur in a number of species. Several studies have demonstrated that male pheromones can communicate aspects of mate quality to females (Thornhill 1992; Droney and Hock 1998; Marco et al. 1998; Martin and Lopez 2000;Reusch et al. 2001;Spurgeon 2003), and there is some evidence for adaptive female choice on the basis of male sex pheromones (Jones and Hamilton 1998; Jones et al. 1998; Jones et al. 2000). However, studies investigating the effect of age on olfactory sexual communication are scarce. In mice, the amount and composition of urinary volatiles has been demonstrated to change with age, and this information is used by conspecifics to discriminate among age groups (Wilson and Harrison 1983; Osada et al. 2003). Age-related changes in the amount and composition of sex pheromone have also been found in male boll weevils (Coleoptera, Curculionidae) (Spurgeon 2003), but the potential importance for female choice has not been investigated.

Females of the European beewolf (Philanthus triangulum, Hymenoptera, Crabronidae) hunt honeybees (Apis mellifera) with which they provision their offspring in underground nest burrows (Strohm and Linsenmair 1997; Strohm and Linsenmair 1999; Strohm and Linsenmair 2000; Strohm and Marliani 2002). Male beewolves establish territories (about 0.25 m^2^ in size), mostly in the vicinity of the females’ nest aggregations, that do not contain any resources for females (Simon-Thomas and Poorter 1972; Strohm 1995). The males mark plants in their territories with the secretion of a cephalic gland and defend them against intruding males in combat flights without physical contact of the opponents (Simon-Thomas and Poorter 1972;Evans and O’Neill 1988; Strohm 1995; Strohm and Lechner 2000; Schmitt et al. 2003). In the field, males can survive for more than four weeks, although the apparent median life span is much shorter since emigrations from a site cannot be dectected (Strohm and Lechner 2000). An individual male can occupy the same territory for several days and up to two weeks (Simon-Thomas and Poorter 1972; Strohm and Lechner 2000). The cephalic gland secretion is likely to serve as a pheromone that attracts receptive females to the male’s territory (Evans and O’Neill 1988). Females of several Philanthus species including our study species have been observed to approach territories of conspecific males in a zigzagging flight pattern from the downwind side, probably orienting towards the windborne cephalic gland components (Evans and O’Neill 1988). Copulations usually occur within the males’ territories (Simon-Thomas and Poorter 1972; Strohm 1995) and seem to be under the control of the females since they can easily repel unwanted mates by virtue of their larger body size (Evans and O’Neill 1988) or refuse copulations by bending their abdomen tip downwards (E.Strohm, pers. obs.). Territories of different males are often found close together, thereby constituting a lek situation in which the females have an ideal opportunity to choose among males (Simon-Thomas and Poorter 1972; Evans and O’Neill 1988). Since the copulation is not preceded by any kind of visual display, female choice appears to be, at least predominantly, based on information obtained from the male marking compounds (E. Strohm and M. Kaltenpoth, unpubl. obs.).

Analyses of head extracts from male European beewolves revealed a complex blend of at least 11 compounds, with (*Z*)-11-eicosen-1-ol as the main component (Schmidt et al. 1990; Schmitt et al. 2003). All of these components are also found in samples of pure cephalic glands in the same relative amounts (Kroiss et al., in prep.) and in extracts from male territories (E. Strohm, T. Schmitt, G. Herzner, J. Kroiss and M. Kaltenpoth, unpubl. data). Although behavioral studies on the biological activity of the components are lacking, these compounds might be important cues for females to assess male quality and choose among potential mates.

Mate choice can be assumed to be of particular importance in the European beewolf. Females most probably mate only once (Evans and O’Neill 1988). Thus, choosing a low-quality male will affect all daughters (due to the haplo-diploid sex determination mechanism, male offspring are not affected, because they do not inherit genes from their mother’s mate). Due to the extraordinary physiological requirements for reproduction including the fact that females have to carry the comparatively heavy prey to their nest in flight, a daughter’s reproductive success heavily depends on her “quality” (Strohm and Linsenmair 1997; Strohm and Daniels 2003). Therefore, “bad” genes from the father might affect a daughter’s ability to hunt honeybees and carry the prey in flight as well as her life span. Thus, female choice for males with “good genes” could strongly influence female fitness. Choosing males who signal either their youth or their high age by virtue of their cephalic gland secretion may be one important factor in this context.

In this study, we analyzed the cephalic gland content of male European beewolves of different age classes to assess the variation of the amount and composition with age. The results are discussed with regard to possible physiological constraints in the production of the compounds and the potential of the cephalic gland secretion as an indicator of male age for female choice.

## Materials and Methods

### Insects and sampling

European beewolves (Philanthus triangulum, Hymenoptera, Crabronidae) were kept in the laboratory at the University of Würzburg. Cocoons with larvae were placed individually in Eppendorf® tubes and kept in boxes with moist sand at 10°C for about eight to nine months of overwintering. Cocoons were then transferred to warm conditions (cycles of 12 hours at 25°C and 12 hours at 22°C) and adult beewolves emerged four to six weeks later. Emerging males were marked individually and were allowed to fly in a climate chamber (2.5 x 1.8 x 2.1 m in size) with 12h light/dark cycles at 25°C/20°C and provided with honey *ad libitum*. Males were caught at different ages and kept overnight in small polystyrol vials (height: 80 mm; diameter: 35 mm) with moist sand and a drop of honey to allow the cephalic glands to be replenished. After anesthetizing the beewolf males with CO_2_, they were killed by freezing and kept frozen (at −20°C) for up to six weeks until extraction of the cephalic gland content and GC-MS analysis. Overall, 107 males were randomly assigned to 13 different age groups, of which the first eleven were spaced four days apart. Groups 1, 5, 9, 13, 21, and 25 comprise only animals of the exact designated age (i.e. 1, 5, 9, 13, 21, and 25 days old at the day when they were frozen, respectively), while groups 17, 29, 33, 37, and 41 include individuals of the designated age and one to three individuals that are up to two days older. Due to the scarcity of very old males, group 47 includes all males between 45 and 49 days, and group 55 includes all males between 50 and 60 days old.

### Gas chromatography - mass spectrometry

Frozen males were decapitated and the heads were incised at both sides to open up the glands. Heads were placed individually in glass vials (4 ml), and 20 μl of a 1g/l solution of octadecane in hexane (equivalent to a final amount of 20 μg of octadecane) was added as an internal standard to each vial to allow quantification of the cephalic gland content. The heads were then submerged in approximately 400 μl distilled hexane and chemicals were extracted for four hours at room temperature. Then each head was removed and the hexane was reduced to about 200 μl by a gentle constant flow of nitrogen. Samples were analyzed by coupled capillary gas chromatography-mass spectrometry (GC-MS) with an Agilent 6890N Series gas chromatograph (Agilent Technologies, www.agilent.com/) coupled to an Agilent 5973 inert mass selective detector. The GC was equipped with a RH-5ms+ fused silica capillary column (J&W, 30 m x 0.25 mm ID; df = 0.25μm; temperature programme: from 60°C to 300°C at 5°C/min, held constant for 1 min at 60°C and for 10 min at 300°C). Helium was used as the carrier gas with a constant flow of 1 ml/min. A split/splitless injector was installed at 250°C in the splitless mode for 60 sec. The electron impact mass spectra were recorded with an ionisation voltage of 70 eV, a source temperature of 230°C and an interface temperature of 315°C. The software MSD ChemStation for Windows was used for data acquisition. The components of the cephalic gland content had already been characterised (Schmitt et al. 2003) and could be unambiguously identified by their retention times and mass spectra.

After GC-MS analysis, the width of the head capsule at the widest point was measured for all males under a dissecting scope (magnification: 40x) with an ocular micrometer scale to control for size differences among the age groups that might account for differences in the amount or composition of the cephalic gland content.

### Statistical analysis

Ten components were included in the analysis and their peaks were integrated with MSD ChemStation software (Agilent Technologies) (Table 1). Using the octadecane peak as an internal standard, the total amount of cephalic gland secretion was calculated and then log_10_-transformed to obtain normally distributed data for statistical analysis. The relative amounts of the ten components were calculated. Two peaks (eicosenol and tricosene) had to be combined for the analysis, since they were not always clearly separated by the GC-MS. With regard to the detection of age-related changes this procedure is conservative. Since the amount of eicosenol by far exceeds that of tricosene (about 10 fold, seeSchmitt et al., 2003), the combined peak is labelled as “eicosenol” in the following. Because the relative amounts constitute compositional data, they were transformed to logcontrasts prior to analysis (Aitchison 1986). The log_10_-transformed absolute amounts of the cephalic gland content and the Aitchison-transformed relative amounts of the components were compared among age groups by one-way ANOVAs with Tukey post hoc tests. Changes in relative amounts of the ten components with age were additionally analysed using linear regression analyses for the males between 5 and 49 days old (see results for reasons to exclude very young and very old males). SPSS 12.0 software was used for the calculations.

**Table 1:**
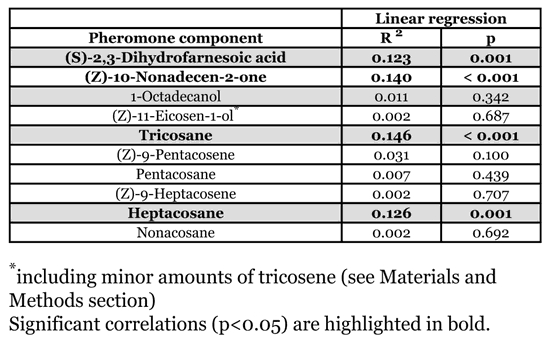
Correlation between age and relative amount of the pheromone components extracted from male Philanthus triangulum between 9 and 49 days of age (Aitchison-transformed).

## Results

### Amount of cephalic gland content

**Figure 1:**
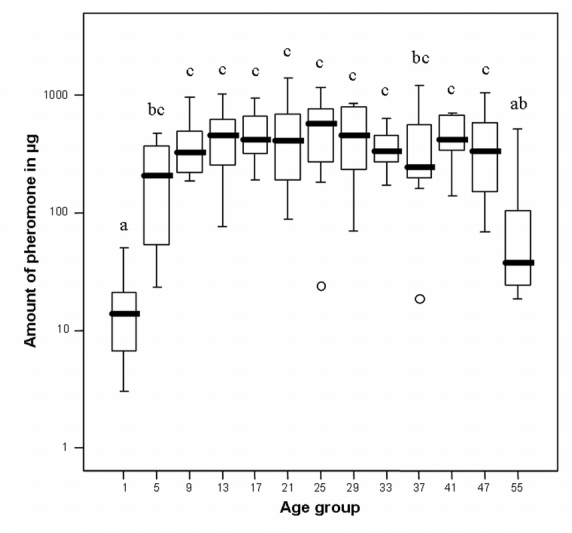
Amount of cephalic gland content extracted from male beewolves of different ages. Bold lines represent medians, boxes comprise the interquartile range, and bars indicate minimum and maximum values, except extreme values, these are represented by circles. Quantities are given in μg on a log_10_-scale. The age groups contain exclusively or predominantly males of the designated age (in days) (exceptions see “Materials and methods” section). Different letters above boxes indicate significant differences between groups (p<0.05). Sample sizes of age groups (from left to right) are: 8, 8, 8, 8, 9, 8, 8, 7, 8, 7, 5, 10, and 11, respectively.

The total extracted amount of compounds in the cephalic gland ranged from 3 to 1400 μg. These values constituted 0.01 to 2.32 % of the respective male’s total body weight. There were significant differences in the total amount of the gland content among the different age groups (ANOVA, F_12, 92_ = 10.1, p < 0.001, Fig. 1). The mean amount increased 13-fold from day one (mean = 17 μg) to day five (mean = 221 μg) and then 1.8 fold to day nine (mean = 408 μg), thereafter remaining more or less constant until the age of 47 days (Figure 1). The oldest males (50–60 days) showed a significant decrease in the amount of compounds extracted from their head glands (mean = 112 μg).

There were no significant size differences (measured as head capsule width) among the age groups that might account for differences in the amount or composition of the gland content (ANOVA, F_12, 94_ = 1,18, p = 0,311).

### Chemical composition of the cephalic gland content

The cephalic secretion of beewolf males of our study population is composed of 11 main components (Schmitt et al. 2003): (S)-2,3-dihydrofarnesoic acid, (Z)-10-nonadecen-2-one, 1-octadecanol, (Z)-11-eicosen-1-ol, (Z)-9-tricosene, tricosane, (Z)-9-pentacosene, pentacosane, (Z)-9-heptacosene, heptacosane, and nonacosane.

**Figure 2:**
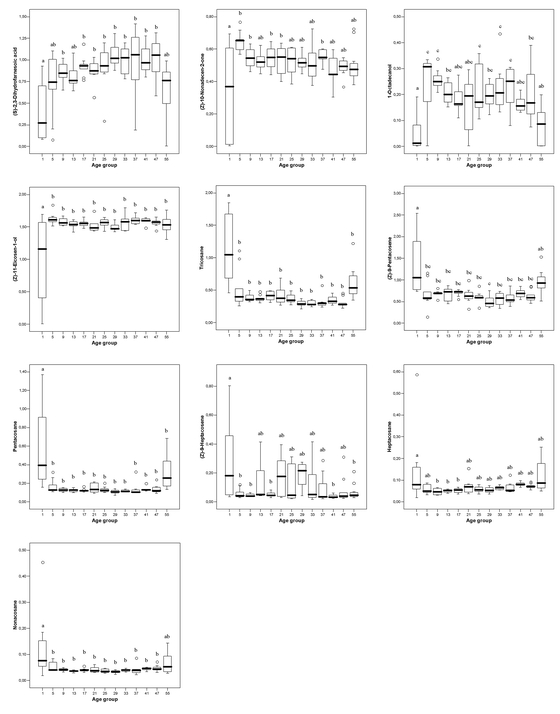
Relative amounts of cephalic gland components extracted from male beewolves of different ages (Aitchison-transformed). Bold lines represent medians, boxes comprise the interquartile range, and bars indicate minimum and maximum values (except extreme values). Extreme values are indicated by circles. (Z)-11-Eicosen-1-ol includes minor amounts of (Z)-9-tricosene. The age groups contain exclusively or predominantly males of the designated age (in days) (exceptions see “Materials and methods” section). Different letters above boxes indicate significant differences between groups (p<0.05). Sample sizes of age groups (from left to right) are: = 8, 9, 8, 9, 9, 8, 8, 7, 8, 7, 5, 10, and 11, respectively.

The chemical composition of the cephalic gland content differed significantly among age groups (Fig. 2). Very young (one day old) males differed from middle-aged males in the relative amounts of most components. Generally, the compounds with functional groups (dihydrofarnesoic acid, nonadecenone, octadecanol, and the peak including eicosenol and tricosene) were present in lower relative quantities in very young males, whereas the alkanes and alkenes (tricosane, pentacosene, pentacosane, heptacosene, heptacosane and nonacosane) were found in larger relative amounts in young males than in the middle-aged males (Fig. 2). The mean relative amounts of dihydrofarnesoic acid, nonadecenone, octadecanol, and eicosenol increased to 254%, 185%, 528%, and 206% from day 1 to day 5, respectively, whereas the mean relative amounts of tricosene, pentacosene, pentacosane, heptacosene, heptacosane, and nonacosane decreased to 26%, 21%, 23%, 19%, 42%, and 45%, respectively, from day 1 to day 5. Although the youngest males showed higher interindividual variation in most of the components than older males, this is unlikely to seriously affect the results of the ANOVAs, because (1) the sample sizes in the groups are roughly equal (especially in the first eight groups), thus reducing effects of unequal variances (Box 1954), and (2) the direction of the effect is the same for all compounds with functional groups. Thus, the low proportion of compounds with functional groups in very young males is consistent for the components with functional groups as opposed to those without functional groups. There was a non-significant trend that the relative amount of compounds with functional groups decreased in very old males (age 50–60 days), whereas the amount of alkanes and alkenes increased (Fig. 2).

The majority of males that are active in the field might be neither very young, nor very old. Thus, to test whether there was an increase or decrease of components among the middle-aged males we conducted a correlation analysis excluding the groups containing very young and very old males. Only the two extreme groups (group 1 and 55) were excluded, because they differed significantly in the total amount (Figure 1) as well as in the chemical composition (Fig. 2) of the cephalic gland secretion, thereby providing evidence for major physiological differences between these groups and the middle-aged males. Among the middle-aged males (5–49 days), the relative amounts of four of the ten compounds were significantly correlated with age: dihydrofarnesoic acid and heptacosane increased with age, whereas nonadecenone and tricosane decreased with age (Table 1). However, these effects were rather small, explaining 12–15 % of the variance of the respective compound.

## Discussion

The results of this study demonstrate that both the total amount and the composition of the male cephalic gland content vary with age in the European beewolf (Philanthus triangulum). This variation might convey information that could be used by females to assess a potential mate’s age and to choose a mate based on its age.

The most dramatic changes in the composition of the males’ cephalic gland content are due to the delay after emergence in the production of components with functional groups (dihydrofarnesoic acid, nonadecenone, octadecanol, and eicosenol) as compared to the alkane/alkene fraction. The onset of production might reflect differences in the metabolic costs of these substances and/or their importance for other functions. The alkanes and alkenes also occur on the cuticle and in the hemolymph of beewolves (Strohm et al., in prep.) and are probably constantly produced by basic metabolic pathways in the oenocytes, as has been shown for other insect taxa (Soroker and Hefetz 2000; Fan et al. 2003). Thus, the production of these compounds does not require specific enzymes and the production costs are therefore expected to be comparatively low.

Dihydrofarnesoic acid, nonadecenone, octadecanol, and eicosenol, however, are only present in the cephalic gland and, thus, specific enzymes are necessary for their production. Preliminary analyses suggest that these components are produced in specialized cells of the large mandibular glands (E. Strohm, G. Herzner, W. Göttler, unpubl. data), so the production additionally requires the formation and maintenance of specialized tissue. Therefore, dihydrofarnesoic acid, nonadecenone, octadecanol, and eicosenol probably inflict higher costs on the males than alkanes and alkenes, and the production of these substances could be limited in very young and very old males due to physiological constraints. This hypothesis is supported by the observation that very young and very old males have significantly smaller amounts of chemicals in the cephalic glands than middle-aged males. That pheromone production can be energetically costly and reduces the subsequent life span of the producer has recently been demonstrated in fruit flies (Johansson et al. 2005).

In very old males, the amount of the components with functional groups decreases, and their cephalic gland composition approaches that of very young males. Although the discrimination of very young and very old males might theoretically pose a problem for female choice, this is unlikely to be the case in the field, because males will very rarely reach an age of 50 days in the field. In fact, the median estimated life span of males in the field was 9 days (lower and upper quartiles: 6 and 18.5), the oldest male was observed for 28 days (Strohm and Lechner 2000). However, these data probably underestimate the real life span of males in the field, because males that emigrated or escaped detection were counted as dead (Strohm & Lechner 2000). Observations in outdoor flight cages provide evidence that males older than 50 days do occur, but are rare even under semi-field conditions (median life span: 17 days, quartiles: 10 and 33 days; Strohm & Lechner 2000). Thus, the age of 50–60 days probably represents the physiological limit of the males’ life span under optimal conditions. Field conditions and territorial activity, i.e. a probably limited availability of food resources and extensive scent marking as well as defense combats, might enhance the effect of age on the composition of the cephalic gland content that has been found under laboratory conditions in this study. Thus, our data probably provide only a lower boundary for the effect of age on the chemical composition of the cephalic gland content.

The changes in cephalic gland composition during the long and probably reproductively most important middle part of a male’s life are less pronounced. However, there are four components that either significantly increase or decrease with age. This might be enough to provide females with some information on the age of a potential mate, especially because the sensitivity of female olfactory receptors and the central nervous processing might be much better than our analytical methods. Furthermore, it is unlikely that females mate only with males of a certain age; they probably rather choose either the youngest or the oldest of the available males, for example by applying a best-of-*n* (i.e. choosing the best mate from a sample of *n* individuals) or a sequential sampling strategy (i.e. choosing the first encountered mate with a quality above a certain critical threshold) (Janetos 1980; Real 1990). Thus, by comparing the scents of a sample of different territories the variation might be sufficient to at least exclude the oldest or the youngest males.

The results of this study indicate that the production of some components of the male beewolf marking secretion vary with age. This might be due to physiological constraints and might therefore provide information on the age of the emitter that might be used for female choice. Behavioral observations and mate choice experiments are necessary to investigate whether females indeed choose mates based on their age, and if so, which components of the male cephalic gland secretion are important for female choice. The results will shed light on the importance of age as a factor for female choice in a species with male chemical signaling. Because of the close association between metabolism and the production of semiochemicals, mate choice based on olfactory cues might be much more common than is currently apparent from the few available studies.
